# Emergency Care Use During Pregnancy and Severe Maternal Morbidity

**DOI:** 10.1001/jamanetworkopen.2024.39939

**Published:** 2024-10-16

**Authors:** Eugene R. Declercq, Chia-Ling Liu, Howard J. Cabral, Ndidiamaka Amutah-Onukagha, Hafsatou Diop, Pooja K. Mehta

**Affiliations:** 1 Boston University School of Public Health, Boston, Massachusetts; 2 Evalogic Services Inc, Newton Centre, Massachusetts; 3 Tufts University School of Medicine, Boston, Massachusetts; 4 Massachusetts Department of Public Health, Boston; 5 Cityblock Health, Brooklyn, New York; 6 Boston University School of Medicine, Boston, Massachusetts

## Abstract

**Importance:**

Pregnant individuals who repeatedly use emergency care during pregnancy represent a population who could be disproportionately vulnerable to harm, including severe maternal morbidity (SMM).

**Objective:**

To explore patterns of unscheduled care visits during pregnancy and ascertain its association with SMM at the time of birth.

**Design, Setting, and Participants:**

This cohort study used data from a statewide database that linked hospital records to births and fetal deaths occurring between October 1, 2002, and March 31, 2020, in Massachusetts. Pregnant individuals experiencing births or fetal deaths during the study period were included. Data analysis was conducted from June 2022 to September 2024.

**Exposure:**

The exposure was 4 or more cases of emergency use, defined as either an emergency department visit or observational stay during pregnancy not resulting in hospital admission. Pregnancy episode was ascertained by subtracting the gestational age at birth from the date of birth.

**Main Outcomes and Measures:**

The outcome of interest was the odds ratio (OR) for SMM at the time of birth. The algorithm includes 20 conditions or procedures (excluding transfusion) identified through *International Classification of Diseases, Ninth Revision* and *International Statistical Classification of Diseases and Related Health Problems, Tenth Revision* codes across the study period.

**Results:**

A total of 774 092 pregnant individuals (mean [SD] age, 31.2 [5.8] years; 16.8% Hispanic, 9.3% non-Hispanic Asian or Pacific Islander, 9.5% non-Hispanic Black, 63.1% non-Hispanic White) with emergency care visits during the pregnancy were included; 31.3% of these individuals had at least 1 visit. Overall, 18.1% had 1 visit and 3.3% had 4 or more visits. Four or more unscheduled visits were common among those younger than age 25 years (8.7%), with Hispanic (5.7%) or non-Hispanic Black (4.9%) race and ethnicity, with public insurance (6.5%), or with a comorbidity (19.0%) or an opioid use–related hospitalization (26.8%) in the year prior to pregnancy. Of those with 4 or more unscheduled visits, 43.8% visited more than 1 hospital during pregnancy. In a multivariable analysis of the likelihood of SMM, those with 4 or more unscheduled visits had an adjusted OR of 1.46 (95% CI, 1.29-1.66) compared with those with 0 visits.

**Conclusions and Relevance:**

This cohort study found that high emergency care use during pregnancy was associated with an increased risk for SMM. With a significant proportion of those with frequent unscheduled visits also using multiple hospitals, solutions that are community-based and integrated across health systems may be most beneficial.

## Key Points

**Question:**

Are those who rely on emergency care during pregnancy a high-risk group for severe maternal morbidity (SMM)?

**Findings:**

In this cohort study of 774 092 pregnant individuals in Massachusetts, 31.3% of this population had an emergency visit during pregnancy, and 3.3% had 4 or more visits. Those with 4 or more visits were more likely to experience SMM at birth.

**Meaning:**

Findings of this study suggest that a group at high risk for SMM can be identified and targeted for preventive interventions.

## Teaser

This cohort study identifies patterns of emergency care visits during pregnancy among individuals in Massachusetts and assesses the association of such visits with severe maternal morbidity.

## Introduction

While multiple studies focus on individuals deemed as high cost, high need,^1,2,3,4,5,6^ implications of high emergency department (ED) use for perinatal health have received limited attention.^7,8^ Pregnancy is a time of intense care use and outcomes that can affect health throughout the life course. Emergency care during pregnancy is increasing in some settings, out of proportion to the prevalence of pregnancy in the population.^9^ Little is known whether unscheduled hospital visits during pregnancy are a meaningful, patient-centered signal of an emerging unmet need associated with pregnancy outcomes. Qualitative research suggests that pregnant individuals with 4 or more unscheduled visits during an index pregnancy have specific unmet clinical and psychosocial needs, including community-based support, tailored medical and prenatal care,^10^ and behavioral health services.^8^ Quantitative studies have examined high perinatal ED use in a Medicaid population in the US^7^ and the association of prepregnancy ED use with severe maternal morbidity (SMM)^11^ and infant hospitalization^12^ in a Canadian province. The latter study found an adjusted risk ratio of 1.37 (95% CI, 1.33-1.42) for SMM in cases where there was an ED encounter 90 days prior to pregnancy.

The objective of the current study was to explore patterns of unscheduled care visits during pregnancy and ascertain its association with SMM at the time of birth. We obtained data from a population-level, longitudinally linked database with a rich set of contextual variables.

## Methods

### Data Source

We used the Massachusetts Pregnancy to Early Life Longitudinal (PELL) Data System for this analysis. Details on this dataset are described elsewhere.^13,14^ The Massachusetts Department of Public Health Institutional Review Board approved this cohort study and deemed it exempt from the informed consent requirement because it was not considered human participant research. We followed the Strengthening the Reporting of Observational Studies in Epidemiology (STROBE) reporting guideline.

Briefly, PELL longitudinally links birth and fetal death records to corresponding maternal hospital encounters (admissions, outpatient stays, and ED visits) in Massachusetts. PELL data are housed at the Massachusetts Department of Public Health and include over 98% of birth certificates and fetal death certificates (≥350 g or ≥20 weeks’ gestation) linked to hospital discharge records from January 1, 1998, through December 31, 2020. PELL records are linked using a deterministic and probabilistic matching program (LinkPro version 3.0; InfoSoft Inc) and are linked to index deliveries according to facility code, medical record number, date of delivery, sex, zip code, and birth weight. To create a longitudinal linkage (before or after delivery), a unique nonmissing encrypted Social Security number and a unique nonmissing combination (concatenated) of hospital numbers and medical record numbers are used. This longitudinal link allows the examination of hospital visits among birthing individuals at any time during the study period, including hospital visits during pregnancy.

### Study Population 

This analysis focused on all individuals with births or fetal deaths in Massachusetts. Data on ED visits became available only in 2002. Therefore, our analytic starting point was October 1, 2002, enabling the identification of unscheduled hospital visits during pregnancy. We chose to end just prior to the start of the COVID-19 pandemic, which profoundly and presumably independently affected ED use,^15^ for a study period from October 1, 2002, to March 31, 2020. The pregnancy period was defined by subtracting the obstetrical estimate of gestational age from the date of birth. Since individuals could have given birth more than once during this period, we limited the analysis to each individual’s most recent pregnancy. Race and ethnicity (Hispanic, non-Hispanic Asian or Pacific Islander, non-Hispanic Black, non-Hispanic White, and non-Hispanic other [including non-Hispanic American Indian and Aleutian]) were self-identified by birthing individuals as part of the parent’s worksheet for the birth certificate. Race and ethnicity data were collected because of their demonstrated relationship with maternal outcomes.

### Statistical Analysis

The core exposure was 4 or more emergency care visits, defined as either an ED or observational stay (OS) visit during pregnancy that was not associated with subsequent hospital admission. We examined demographic and socioeconomic characteristics of pregnant individuals available on birth and fetal death certificates as well as a validated measure of prenatal care adequacy^16^ by the number of emergency hospital visits and timing of emergency visits during pregnancy. Throughout the analysis, we referred to scheduled obstetric outpatient prenatal care as *prenatal visits* to distinguish them from ED and OS visits, which occur in EDs or hospital obstetric triage units and are more likely to be patient-initiated and unscheduled. All demographic factors were obtained from the birth or fetal death certificate data.

Due to the literature suggesting that opioid use is a specific factor in unscheduled visits, we identified from the dataset hospitalizations in the year prior to pregnancy associated with a standard measure of comorbidities^17,18^ (exclusive of opioid use) and hospital visits related to opioid use.^19^ We also included a measure of prepregnancy diabetes from the birth certificate. While there is no consensus definition of high use of prenatal emergency care, building on the largest prior study of pregnant individuals with high utilization,^7^ we categorized 4 or more unscheduled hospital visits as high use. To ascertain whether those with multiple unscheduled visits during pregnancy were experiencing fragmentation in care, which might affect solutions, we examined the number of different hospitals used and the association between the number of unique hospitals visited and SMM. The analysis was conducted from June 2022 to September 2024 using SAS, version 9.4 (SAS Institute Inc).

There have been multiple iterations of the SMM measure.^20,21,22^ To enhance comparability over time, we examined instances of SMM during the delivery hospitalization using the algorithm developed as part of an interagency collaboration between the Health Resources and Services Administration, Centers for Disease Control and Prevention, Agency for Healthcare Research and Quality, and Alliance for Innovation on Maternal Health (version 07-01-2021).^23^ Since the validity of the coding of transfusion has been excluded from national SMM measures due to poor specificity in the absence of other SMM indicators,^20,24^ we excluded transfusion and focused on 20 SMM conditions or procedures identified through *International Classification of Diseases, Ninth Revision* and *International Statistical Classification of Diseases and Related Health Problems, Tenth Revision* codes across the study period.^13,25,26^ Given that outcomes were rare, we used multiple logistic regression analysis as the primary means to assess the association of hospital visits with SMM in models that were unadjusted and adjusted for relevant covariates chosen a priori based on the literature. We generated unadjusted and adjusted odds ratios (AORs) with 95% CIs from these models.

## Results

There were 1 248 534 deliveries in Massachusetts between October 1, 2002, and March 31, 2020. We excluded those with missing data on gestational age (n = 2028), missing delivery records needed to define SMM (n = 12 176), or missing data on covariates (n = 15 755). We included both singleton and multiple births. In cases when the dataset indicated an individual gave birth more than once, we chose the most recent birth, thus excluding an additional 444 483 deliveries. The final study sample comprised 774 092 birthing individuals (mean [SD] age, 31.2 [5.8] years; 129 894 [16.8%] Hispanic, 72 134 [9.3%] non-Hispanic Asian or Pacific Islander, 73 821 [9.5%] non-Hispanic Black, 488 244 [63.1%] non-Hispanic White, and 9999 [1.3%] non-Hispanic other individuals) (Table 1). Of these individuals, 0.7% experienced SMM and 99.3% did not (eFigure 1 in Supplement 1).

**Table 1.** Characteristics of Individuals With Emergency Hospital Visits During Pregnancy in Massachusetts From October 1, 2002, to March 31, 2020

Characteristic	No. (%)					
	Individuals (N = 774 092)^a^	Antenatal ED or OS visit				
		0 (n = 532 012)	1 (n = 140 453)	2 (n = 52 880)	3 (n = 22 976)	≥4 (n = 25 771)
Age, y						
≤24	110 697 (14.3)	54 191 (49.0)	26 026 (23.5)	13 714 (12.4)	7147 (6.5)	9619 (8.7)
25-29	166 050 (21.5)	103 238 (62.2)	34 220 (20.6)	14 451 (8.7)	6522 (3.9)	7619 (4.6)
30-34	262 233 (33.9)	194 229 (74.1)	43 394 (16.5)	14 004 (5.3)	5403 (2.1)	5203 (2.0)
≥35	235 112 (30.4)	180 354 (76.7)	36 813 (15.7)	10 711 (4.6)	3904 (1.7)	3330 (1.4)
Race and ethnicity^b^						
Hispanic	129 894 (16.8)	74 435 (57.3)	28 346 (21.8)	13 106 (10.1)	6543 (5.0)	7464 (5.7)
Non-Hispanic Asian or Pacific Islander	72 134 (9.3)	60 107 (83.3)	8876 (12.3)	2095 (2.9)	642 (0.9)	414 (0.6)
Non-Hispanic Black	73 821 (9.5)	42 853 (58.0)	16 542 (22.4)	7406 (10.0)	3421 (4.6)	3599 (4.9)
Non-Hispanic White	488 244 (63.1)	348 085 (71.3)	84 771 (17.4)	29 452 (6.0)	12 016 (2.5)	13 920 (2.9)
Non-Hispanic other^c^	9999 (1.3)	6532 (65.3)	1918 (19.2)	821 (8.2)	354 (3.5)	374 (3.7)
Parity						
1	267 640 (34.6)	181 529 (67.8)	49 350 (18.4)	18 992 (7.1)	8394 (3.1)	9375 (3.5)
2	310 077 (40.1)	222 343 (71.7)	53 165 (17.1)	18 566 (6.0)	7685 (2.5)	8318 (2.7)
≥3	196 375 (25.4)	128 140 (65.3)	37 938 (19.3)	15 322 (7.8)	6897 (3.5)	8078 (4.1)
Nativity						
US, including Puerto Rico	536 770 (69.3)	358 081 (66.7)	100 628 (18.7)	39 266 (7.3)	17 589 (3.3)	21 206 (4.0)
Born outside the US	237 322 (30.7)	173 931 (73.3)	39 825 (16.8)	13 614 (5.7)	5387 (2.3)	4565 (1.9)
Maternal educational level						
≤High school diploma or GED certificate	274 848 (35.5)	155 399 (56.5)	60 698 (22.1)	28 047 (10.2)	13 518 (4.9)	17 186 (6.3)
Some college	145 913 (18.8)	89 679 (61.5)	31 022 (21.3)	13 039 (8.9)	5986 (4.1)	6187 (4.2)
Bachelor’s and/or graduate degree	353 331 (45.6)	286 934 (81.2)	48 733 (13.8)	11 794 (3.3)	3472 (1.0)	2398 (0.7)
Marital status						
Married	528 044 (68.2)	401 634 (76.1)	83 708 (15.9)	25 214 (4.8)	9094 (1.7)	8394 (1.6)
Unmarried	246 048 (31.8)	130 378 (53.0)	56 745 (23.1)	27 666 (11.2)	13 882 (5.6)	17 377 (7.1)
Payer for birth						
Private	427 803 (55.3)	334 033 (78.1)	65 760 (15.4)	17 844 (4.2)	5788 (1.4)	4378 (1.0)
Public or free care	329 330 (42.5)	183 630 (55.8)	72 828 (22.1)	34 546 (10.5)	17 051 (5.2)	21 275 (6.5)
Self-pay	16 959 (2.2)	14 349 (84.6)	1865 (11.0)	490 (2.9)	137 (0.8)	118 (0.7)
Comorbidity (excluding opioid use) 1 y prior to pregnancy^d^						
No	746 998 (96.5)	523 383 (70.1)	134 078 (17.9)	48 636 (6.5)	20 278 (2.7)	20 623 (2.8)
Yes	27 094 (3.5)	8629 (31.8)	6375 (23.5)	4244 (15.7)	2698 (10.0)	5148 (19.0)
Opioid use 1 y prior to pregnancy^d^						
No	770 303 (99.5)	531 100 (68.9)	139 602 (18.1)	52 261 (6.8)	22 585 (2.9)	24 755 (3.2)
Yes	3789 (0.5)	912 (24.1)	851 (22.5)	619 (16.3)	391 (10.3)	1016 (26.8)
Prepregnancy diabetes^e^						
No	763 510 (98.6)	525 667 (68.8)	138 288 (18.1)	51 919 (6.8)	22 542 (3.0)	25 094 (3.3)
Yes	10 582 (1.4)	6345 (60.0)	2165 (20.5)	961 (9.1)	434 (4.1)	677 (6.4)
Adequacy of PNC						
None	2990 (0.4)	2132 (71.3)	470 (15.7)	189 (6.3)	77 (2.6)	122 (4.1)
Inadequate	72 846 (9.4)	48 617 (66.7)	13 090 (18.0)	5376 (7.4)	2491 (3.4)	3264 (4.5)
Intermediate	49 900 (6.4)	33 039 (66.2)	9551 (19.1)	3713 (7.4)	1682 (3.4)	1916 (3.8)
Adequate	318 385 (41.1)	230 797 (72.5)	53 712 (16.9)	18 657 (5.9)	7609 (2.4)	7641 (2.4)
Adequate plus	312 338 (40.3)	205 487 (65.8)	60 469 (19.4)	23 738 (7.6)	10 526 (3.4)	12 119 (3.9)
Missing data	17 633 (2.3)	11 936 (67.7)	3179 (18.0)	1213 (6.9)	607 (3.4)	698 (4.0)
Gestational age at delivery, wk						
<32	10 666 (1.4)	6269 (58.8)	2390 (22.4)	1025 (9.6)	445 (4.2)	536 (5.0)
32-36	54 178 (7.0)	33 130 (61.2)	11 139 (20.6)	4703 (8.7)	2275 (4.2)	2926 (5.4)
37-38	173 727 (22.4)	115 320 (66.4)	32 887 (18.9)	12 891 (7.4)	5768 (3.3)	6880 (4.0)
>38	535 521 (69.2)	377 275 (70.5)	94 037 (17.6)	34 273 (6.4)	14 513 (2.7)	15 423 (2.9)
Year of most recent birth						
2002-2005	131 001 (16.9)	91 268 (69.7)	24 274 (18.5)	8423 (6.4)	3406 (2.6)	3629 (2.8)
2006-2008	120 609 (15.6)	81 954 (68.0)	22 771 (18.9)	8358 (6.9)	3498 (2.9)	4028 (3.3)
2009-2011	116 886 (15.1)	78 325 (67.0)	22 220 (19.0)	8486 (7.3)	3659 (3.1)	4185 (3.6)
2012-2014	122 900 (15.9)	83 879 (68.3)	22 331 (18.2)	8750 (7.1)	3773 (3.1)	4166 (3.4)
2015-2017	143 433 (18.5)	98 811 (68.9)	25 388 (17.7)	9753 (6.8)	4504 (3.1)	4963 (3.5)
2018-2020	139 263 (18.0)	97 749 (70.2)	23 466 (16.9)	9108 (6.5)	4136 (3.0)	4805 (3.5)

Abbreviations: ED, emergency department; GED, General Educational Development; OS, observational stay; PNC, prenatal care.

^a^ Percentages in this column represent proportions of the total study population (774 092).

^b^ Self-identified by birthing individuals in the parent’s worksheet of the birth certificate.

^c^ Non-Hispanic other includes non-Hispanic American Indian and Aleutian.

^d^ Based on nondelivery hospitalization records.

^e^ Based on birth certificate and fetal death vitals and hospital discharge delivery records.

Of 774 092 birthing individuals included in the analysis,31.3% had an unscheduled hospital visit (ED or OS) during pregnancy (24.8% ED; 10.5% OS) (Table 2). Overall, 18.1% had 1 unscheduled hospital visit, 6.8% had 2, 3.0% had 3, and a total of 3.3% had 4 or more.

**Table 2.** Distribution of Unscheduled Hospital Visits During Pregnancy in Massachusetts From October 1, 2002, to March 31, 2020

No. of visits	Visit type, No. (%) (N = 774 092)		
	ED	OS	ED or OS (analytic group)
0	582 434 (75.2)	692 488 (89.5)	532 012 (68.7)
Any visit	191 658 (24.8)	81 604 (10.5)	242 080 (31.3)
1	117 926 (15.2)	60 068 (7.8)	140 453 (18.1)
2	40 204 (5.2)	13 619 (1.8)	52 880 (6.8)
3	16 586 (2.1)	4505 (0.6)	22 976 (3.0)
4	7690 (1.0)	1745 (0.2)	11 156 (1.4)
5	3943 (0.5)	843 (0.1)	6041 (0.8)
≥6	5309 (0.7)	824 (0.1)	8574 (1.1)

Abbreviations: ED, emergency department; OS, observational stay.

Table 1 presents selected demographic characteristics of the sample by number of unscheduled hospital visits. High emergency care use was more common among younger individuals, with 8.7% (9619 of 110 697) of those younger than age of 25 years having 4 or more unscheduled visits compared with only 1.4% (3330 of 235 112) of those 35 years or older. Highest emergency care use was found among Hispanic individuals (5.7% [7464 of 12 894]), followed by non-Hispanic Black individuals (4.9% [3599 of 73 821]), non-Hispanic White individuals (2.9% [13 920 of 488 244]), and non-Hispanic Asian individuals (0.6% [414 of 72 134]). Individuals with public insurance were more likely to have 4 or more unscheduled visits compared with those with private insurance (6.5% [21 275 of 329 330] vs 1.0% [4378 of 427 803]). Nineteen percent of individuals (5148 of 27 094) with a comorbidity excluding opioid use in the year prior to pregnancy demonstrated a high number of unscheduled visits, and 26.8% (1016 of 3789) of those with a hospital visit associated with opioid use in the year prior to pregnancy had 4 or more unscheduled hospital visits during pregnancy. High emergency care use was also more common among those with lower educational levels, who were US born, who were unmarried, who gave birth at less than 36 weeks’ gestation, and with preexisting diabetes. Unscheduled visits were most likely to occur during the first 8 weeks of pregnancy and peaked again between 32 and 36 weeks’ gestation, especially among OS visits (eFigure 2 in Supplement 1).

In an exploratory analysis informed by prior literature, we examined the prevalence of diagnoses, grouping by trimester and association with SMM. We found that among individuals with SMM, there were more emergency visits for hematologic and cardiovascular conditions than among those without SMM. For example, during the prenatal period, among those with SMM, there were higher rates of emergency visits for hematologic (6.4% [95% CI, 5.3%-7.4%] vs 2.0% [95% CI, 2.0%-2.1%]) and cardiovascular conditions (8.6% [95% CI, 7.4%-9.8%] vs 3.6% [95% CI, 3.6%-3.7%]) than among those without SMM (eTable 1 in Supplement 1). There was no clear pattern between adequacy of prenatal care and high unscheduled emergency care use, and no clear temporal pattern was observed over the 18-year study period. Therefore, we excluded prenatal care adequacy and year of birth from the multivariable model. Only 2990 of 774 092 individuals (0.4%) were identified as receiving no prenatal care, and 2132 (71.3%) of those individuals also had no unscheduled visits.

**Figure 1.**
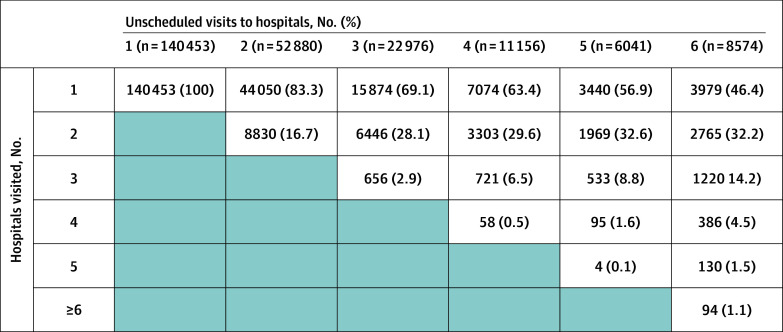
Unscheduled Hospital Visits During Pregnancy by Number of Different Hospitals Used in Massachusetts From October 1, 2002, to March 31, 2020 Shaded areas indicate impossible options (eg, if someone has only 1 prenatal visit they could only go to 1 hospital).

**Figure 2.**
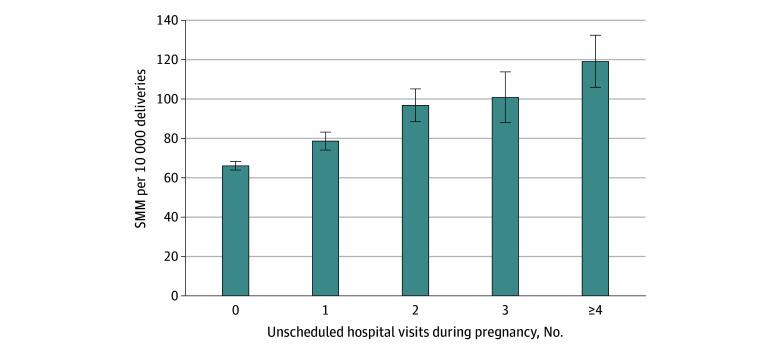
Severe Maternal Morbidity (SMM) by Unscheduled Hospital Visits During Pregnancy in Massachusetts Between October 1, 2002, and March 31, 2020 Based on most recent birth experience (N = 774 092). Error bars represent 95% CIs.

We examined the number of different hospitals that were visited during the prenatal period and found that 11 278 of 25 771 individuals (43.8%) with 4 or more visits used more than 1 hospital and 3241 (12.6%) visited 3 or more hospitals (Figure 1). The rate of SMM increased with the number of hospitals visited during pregnancy, from 85.9 (95% CI, 82.1-89.9) per 10 000 deliveries among those going to 1 hospital to 141.1 (95% CI, 106.5-183.3) per 10 000 deliveries for those receiving emergency care in 3 or more hospitals. The bivariate association between number of unscheduled visits and SMM is shown in Figure 2, with a steady increase in SMM rate from 0 visits (66.2 [95% CI, 64.0-68.3] per 10 000 deliveries) through 4 or more visits (119.1 [95% CI, 105.9-132.4] per 10 000 deliveries).

We examined the association between high emergency care use and SMM in unadjusted and adjusted logistic regression models, controlling for multiple demographic and health conditions (Table 3). In the adjusted model, the rate of SMM increased with the number of unscheduled hospital visits. Among those with 3 unscheduled prenatal hospital visits, the AOR for experiencing SMM was 1.31 (95% CI, 1.14-1.50), and the AOR for those with 4 or more visits was 1.46 (95% CI, 1.29-1.66) compared with those who had 0 visits. This association existed despite the model including a number of factors independently associated with severe morbidity, including age 35 years or older (AOR, 1.55; 95% CI, 1.43-1.67), experiencing a comorbidity in the year prior to pregnancy (AOR, 1.58; 95% CI, 1.42-1.77), being of non-Hispanic Black race and ethnicity (AOR, 1.81; 95% CI, 1.67-1.97), having prepregnancy diabetes (AOR, 1.57; 95% CI, 1.36-1.81), or having an opioid use–related hospitalization in the year prior to pregnancy (AOR, 1.86; 95% CI, 1.44-2.42). Gestational age was associated with SMM, particularly for 10 666 of 774 092 individuals (1.4%) who delivered at less than 32 weeks’ gestation (AOR, 8.25; 95% CI, 7.47-9.12).

**Table 3.** Unadjusted and Adjusted Odds Ratios for Severe Maternal Morbidity by Unscheduled Hospital Visits During Pregnancy and Demographic and Medical Characteristics in Massachusetts From October 1, 2002, to March 31, 2020

	20 SMM conditions at delivery, No. (%)		20 SMM conditions at delivery per 10 000 deliveries	OR (95% CI)	
	Yes	No		Crude^a^	Adjusted^a^
Total	5676	768 416	73.3	NA	NA
No. of ED or OS visits					
0	3520 (62.0)	528 492 (68.8)	66.2	1 [Reference]	1 [Reference]
1	1105 (19.5)	139 348 (18.1)	78.7	1.19 (1.11-1.27)	1.10 (1.03-1.18)
2	512 (9.0)	52 368 (6.8)	96.8	1.47 (1.34-1.61)	1.30 (1.18-1.44)
3	232 (4.1)	22 744 (3.0)	101.0	1.53 (1.34-1.75)	1.31 (1.14-1.50)
≥4	307 (5.4)	25 464 (3.3)	119.1	1.81 (1.61-2.04)	1.46 (1.29-1.66)
Gestational age at delivery, wk					
<32	492 (8.7)	10 174 (1.3)	461.3	10.07 (9.12-1.11)	8.25 (7.47-9.12)
32-36	1292 (22.8)	52 886 (6.9)	238.5	5.09 (4.75-5.44)	4.51 (4.21-4.83)
37-38	1332 (23.5)	172 395 (22.4)	76.7	1.61 (1.51-1.72)	1.54 (1.44-1.64)
>38	2560 (45.1)	532 961 (69.4)	47.8	1 [Reference]	1 [Reference]
Age, y					
≤24	648 (11.4)	110 049 (14.3)	58.5	0.87 (0.79-0.96)	0.74 (0.66-0.81)
25-29	1112 (19.6)	164 938 (21.5)	67.0	1 [Reference]	1 [Reference]
30-34	1750 (30.8)	260 483 (33.9)	66.7	1.00 (0.92-1.08)	1.14 (1.06-1.24)
≥35	2166 (38.2)	232 946 (30.3)	92.1	1.38 (1.28-1.48)	1.55 (1.43-1.67)
Race and ethnicity^b^					
Hispanic	1042 (18.4)	128 852 (16.8)	80.2	1.30 (1.21-1.39)	1.25 (1.15-1.36)
Non-Hispanic White	3024 (53.3)	485 220 (63.1)	61.9	1 [Reference]	1 [Reference]
Non-Hispanic Black	1013 (17.8)	72 808 (9.5)	137.2	2.23 (2.08-2.40)	1.81 (1.67-1.97)
Non-Hispanic Asian or Pacific Islander	523 (9.2)	71 611 (9.3)	72.5	1.17 (1.07-1.29)	1.06 (0.96-1.18)
Non-Hispanic other^c^	74 (1.3)	9925 (1.3)	74.0	1.20 (0.95-1.51)	1.10 (0.87-1.39)
Parity					
1	2430 (42.8)	265 210 (34.5)	90.8	1 [Reference]	1 [Reference]
2	1727 (30.4)	308 350 (40.1)	55.7	0.61 (0.58-0.65)	0.61 (0.57-0.65)
≥3	1519 (26.8)	194 856 (25.4)	77.4	0.85 (0.80-0.91)	0.69 (0.64-0.74)
Nativity					
US, including Puerto Rico	3616 (63.7)	533 154 (69.4)	67.4	1 [Reference]	1 [Reference]
Born outside the US	2060 (36.3)	235 262 (30.6)	86.8	1.29 (1.22-1.36)	1.13 (1.06-1.21)
Educational level					
≤High school diploma or GED certificate	2027 (35.7)	272 821 (35.5)	73.7	1.04 (0.98-1.11)	0.89 (0.82-0.96)
Some college	1148 (20.2)	144 765 (18.8)	78.7	1.11 (1.04-1.19)	0.95 (0.87-1.02)
Bachelor’s and/or graduate degree	2501 (44.1)	350 830 (45.7)	70.8	1 [Reference]	1 [Reference]
Marital status					
Married	3712 (65.4)	524 332 (68.2)	70.3	1 [Reference]	1 [Reference]
Unmarried	1964 (34.6)	244 084 (31.8)	79.8	1.14 (1.08-1.20)	0.97 (0.90-1.04)
Payer for birth					
Private	2751 (48.5)	425 052 (55.3)	64.3	1 [Reference]	1 [Reference]
Public or free care	2716 (47.9)	326 614 (42.5)	82.5	1.29 (1.22-1.36)	1.20 (1.12-1.29)
Self-pay	209 (3.7)	16 750 (2.2)	123.2	1.93 (1.67-2.22)	1.56 (1.35-1.80)
Comorbidity (excluding opioid use) 1 y prior to pregnancy^d^					
No	5265 (92.8)	741 733 (96.5)	70.5	1 [Reference]	1 [Reference]
Yes	411 (7.2)	26 683 (3.5)	151.7	2.17 (1.96-2.40)	1.58 (1.42-1.77)
Opioid use 1 y prior to pregnancy^d^					
No	5613 (98.9)	764 690 (99.5)	72.9	1 [Reference]	1 [Reference]
Yes	63 (1.1)	3726 (0.5)	166.3	2.30 (1.79-2.96)	1.86 (1.44-2.42)
Chronic diabetes^e^					
No	5463 (96.2)	758 047 (98.7)	71.6	1 [Reference]	1 [Reference]
Yes	213 (3.8)	10 369 (1.3)	201.3	2.85 (2.48-3.27)	1.57 (1.36-1.81)

Abbreviations: ED, emergency department; GED, General Educational Development; NA, not applicable; OR, odds ratio; OS, observational stay; SMM, severe maternal morbidity.

^a^ Logistic regressions were used to estimate the associations between 20 SMM conditions at delivery and covariates in the table.

^b^ Self-identified by birthing individuals in the parent’s worksheet of the birth certificate.

^c^ Non-Hispanic other includes non-Hispanic American Indian and Aleutian.

^d^ Based on hospital discharge nondelivery records.

^e^ Based on birth and fetal death certificate vital records and hospital delivery records.

Individuals with multiple births had higher rates of SMM^27^ than individuals with singleton births, regardless of the number of emergency visits, but not higher rates of emergency visits. The increase of prenatal ED or OS visits was not associated with higher SMM rate at delivery. However, the increase of prenatal ED or OS visits was associated with a higher SMM rate at delivery among individuals with multiple births or prior SMM. Because the proportions of individuals with multiple births (2.8%) or prior SMM (0.4%) were small, their ability to change the overall results was minimal (eTable 2 in Supplement 1).

## Discussion

To our knowledge, this study was the first US-based research to report on the association of 4 or more emergency care visits during pregnancy with having SMM. We found that 31.3% of birthing individuals in Massachusetts had at least 1 unscheduled visit during pregnancy, and 3.3% had at least 4 visits. These individuals were more likely to be younger, have a lower educational level, be of Hispanic or non-Hispanic Black race and ethnicity, have public insurance, and have a comorbidity or opioid use in the year prior to pregnancy. A notable segment of this group also visited multiple hospitals for care during pregnancy.

Prior studies of high unscheduled hospital use during and before pregnancy focused on different populations, geographic areas, and time frames. In a study of Medicaid members in North Carolina, Vladutiu et al^7^ found that 57.5% of participants had an encounter with emergency care or an obstetrical triage unit during pregnancy and that 18.1% had 4 or more encounters. The present study confirmed the previous findings that emergency visits were most likely to occur in the first 8 weeks and the last 8 weeks of pregnancy. In a study in Ontario, Canada, of ED encounters in the 90 days prior to pregnancy, Varner et al^11^ found that 9.7% of the sample had a prepregnancy ED encounter and those with such a visit were more likely to experience SMM (adjusted risk ratio, 1.37; 95% CI, 1.33-1.42), in line with our study finding of a greater likelihood of SMM (AOR, 1.46; 95% CI, 1.29-1.66). Moreover, qualitative research suggests that the distinct populations of (1) pregnant individuals frequently turning to emergency prenatal care and (2) those who do not use hospital or outpatient prenatal care at all due to barriers can represent the rational preferences of distinct, particular subgroups within demographically similar populations. These preferences are associated with burden of social isolation, neighborhood or geographic factors, history of trauma, and/or complex illness.^8^

The findings of this study go beyond prior single-site and single-payer analyses reporting that birthing people with multiple unscheduled visits during pregnancy are more likely to experience poor outcomes. First, by examining visits across multiple hospitals, insurance types, care settings, and years, the study more accurately reflects population-level use and needs. Second, by establishing that there was no association between adequate prenatal care and emergency visits despite regional patterns of broader outpatient access and insurance coverage during the study period,^28^ the study reveals that traditional prenatal care may not adequately meet the needs of a subgroup of pregnant individuals more likely to experience SMM. Third, the study used historical categories of race and ethnicity as captured on birth certificates to confirm racial disparities in who uses the ED during pregnancy. The adjusted findings in context with qualitative data and systematic reviews suggest potential manifestations of structural racism. Such manifestations include structural barriers that systematically disadvantage racially and ethnically minoritized people and benefit White people in housing, neighborhood safety, education, employment benefits, and access to culturally acceptable prenatal care, in addition to interpersonal and institutional racism.^8,29,30^ All of these factors have been shown to affect outpatient prenatal care use and, independently, pregnancy outcome. Unscheduled emergency care may represent unmet clinical and psychosocial need, growing vulnerability to adverse outcomes experienced in the hospital, and specific missed opportunities for meaningful care and support.^10^

We believe that our study draws an important connection between emergency care use and SMM as a marker of the relevance of these unmet needs to a measurable, consequential clinical outcome valued by patients, communities, payers, regulators, and advocates alike. Beyond SMM, approximately half of all decedents in 1 study of pregnancy-associated mortality had unscheduled care within a month of their death,^31^ while another study found 69.9% of those with a pregnancy associated death had a hospital visit between the birth and their hospitalization at death.^26^

Payers and innovators are increasingly able to analyze hospital and ED data in real time to offer alternate modalities of care to individuals demonstrating unmet needs during pregnancy, but meaningful action is limited by siloed communication and fragmentation between hospitals, outpatient prenatal care, and networks of community-based support. Strategies such as perinatal community health worker or doula support,^32^ community-based prenatal care with focused referral for high-acuity needs,^33^ referral or cash assistance to mitigate social determinants of health, nurse home visiting program,^34^ virtual behavioral health support,^35^ and evidence-based care for substance use disorder^36,37^ all show promise but are offered piecemeal or via single institutions.

### Strengths and Limitations

Study strengths include a longitudinal population-based data source, which linked vital records and hospital discharge data. This feature enabled us to track hospitalizations over time, assess results across groups with different levels of medical and social risks, and test associations with a critical clinical outcome of interest.

This study also has several limitations. The demographics of Massachusetts deliveries are not comparable to those of US births, with fewer non-Hispanic Black and Hispanic births and a higher proportion of births to older individuals than in some states.^13^ There is no standard definition for high emergency care use during pregnancy; hence, we focused on ED and OS visits not associated with hospital admission and selected 4 or more visits as a threshold, as other studies have done,^7^ after confirming that this represented a meaningful percentage of the study sample. Furthermore, while many emergency visits during pregnancy take place in obstetric triage units attached to labor and delivery units after 20 weeks of pregnancy, we were not able to identify specific obstetric triage visits in the PELL dataset, which is composed of discharge records and not revenue codes or claims. Some hospitals code obstetric triage visits as emergency visits, but in our experience, other facilities code them as OS or even outpatient visits.^38^ This means that we may have underestimated emergency care use during pregnancy and its association with SMM. The SMM measure itself has undergone several changes over the years,^22^ and the SMM rates in this study may not be comparable with those in national studies, particularly those that include transfusions in their SMM measure. We could not capture encounters at hospitals outside of Massachusetts, nor could we account for the potential for residual or unmeasured confounding. The administrative dataset does not capture all clinical factors during the delivery, and we could not incorporate health system structure, policies, resources, and staffing models, which may affect outcomes. We combined ED and OS visits. However, when analyzed separately, we did not find significant differences in SMM rates in part because of the small number of those with more than 1 OS visit (2.8%). Additionally, the finding that multiple hospitals were visited for unscheduled care may be affected by Massachusetts having a relatively high concentration of hospitals.

## Conclusions 

The findings of this study remind us that pregnant individuals are knocking on hospital doors seeking assistance. Despite increased access to and use of scheduled outpatient prenatal care and increasing expenditures on hospital-based obstetric care, inequitable maternal and neonatal outcomes are persistent. To prevent morbidity or worse, we urgently need integrated responses to granular signals of need during the critical period of pregnancy.

## Notes

Supplement 1. **eTable 1.** Conditions Associated With Unscheduled Visits During Pregnancy, Massachusetts, 2002-2020 **eTable 2.** SMM (per 10,000 Deliveries) Among Those With Either an Emergency Department (ED) Visit or Observational Stay (OBS) Using Women’s Most Recent Delivery, Massachusetts (10/1/2002-3/31/2020) **eFigure 1.** Study Sample **eFigure 2.** Timing of Unscheduled Prenatal Hospital Utilization by Prenatal Month, Massachusetts, 10/1/2002-3/31/2020

Supplement 2.
Data Sharing Statement
