# Supplementary material for: Emergency Care Use During Pregnancy and Severe Maternal Morbidity
Source: JAMA Netw Open. 2024 Oct 16;7(10):e2439939. doi: 10.1001/jamanetworkopen.2024.39939 (PMC11581629; doi:10.1001/jamanetworkopen.2024.39939)
Supplement: Supplement 1. — eTable 1. Conditions Associated With Unscheduled Visits During Pregnancy, Massachusetts, 2002-2020 eTable 2. SMM (per 10,000 Deliveries) Among Those With Either an Emergency Department (ED) Visit or Observational Stay (OBS) Using Women’s Most Recent Delivery, Massachusetts (10/1/2002-3/31/2020) eFigure 1. Study Sample eFigure 2. Timing of Unscheduled Prenatal Hospital Utilization by Prenatal Month, Massachusetts, 10/1/2002-3/31/2020 [file jamanetwopen-e2439939-s001.pdf]

Supplemental Online Content

Declercq ER, Liu C-L, Cabral HJ, Amutah-Onukagha N, Diop H, Mehta PK. Emergency care use during pregnancy and severe maternal morbidity. *JAMA Netw Open*. 2024;7(10):e2439939. doi:10.1001/jamanetworkopen.2024.39939

eTable 1. Conditions Associated With Unscheduled Visits During Pregnancy, Massachusetts, 2002-2020

eTable 2. SMM (per 10,000 Deliveries) Among Those With Either an Emergency Department (ED) Visit or Observational Stay (OBS) Using Women's Most Recent Delivery, Massachusetts (10/1/2002-3/31/2020)

eFigure 1. Study Sample

eFigure 2. Timing of Unscheduled Prenatal Hospital Utilization by Prenatal Month, Massachusetts, 10/1/2002-3/31/2020

This supplemental material has been provided by the authors to give readers additional information about their work.

© 2024 Declercq ER et al. *JAMA Network Open*

eTable 1. Conditions Associated with Unscheduled Visits during Pregnancy, Massachusetts, 2002-2020

	Prenatal				First trimester				Second trimester				Third trimester			
	SMM (n=2,156)		non-SMM (n=239,924)		SMM (n=1,363)		non-SMM (n=140,514)		SMM (n=866)		non-SMM (n=83,990)		SMM (n=702)		non-SMM (n=89,383)	
	n	%	n	%	n	%	n	%	n	%	n	%	n	%	n	%
Neuro/Psychiatric	261	12.1	22,971	9.6	164	12.0	14,819	10.5	89	10.3	6,772	8.1	56	8.0	4,579	5.1
Respiratory/Pulmonary	330	15.3	33,231	13.9	175	12.8	17,702	12.6	138	15.9	11,926	14.2	64	9.1	6,928	7.8
Musculoskeletal	304	14.1	27,085	11.3	185	13.6	13,610	9.7	103	11.9	8,861	10.6	50	7.1	6,212	6.9
Gastrointestinal/Digestive	208	9.6	21,455	8.9	119	8.7	12,028	8.6	72	8.3	6,578	7.8	31	4.4	4,218	4.7
Endocrine	232	10.8	19,673	8.2	144	10.6	11,316	8.1	73	8.4	5,365	6.4	45	6.4	4,374	4.9
Cardiovascular	185	8.6	8,716	3.6	98	7.2	4,691	3.3	61	7.0	2,563	3.1	46	6.6	1,951	2.2
Renal/Urinary	297	13.8	32,427	13.5	205	15.0	22,253	15.8	69	8.0	7,441	8.9	38	5.4	4,605	5.2
Hematologic	137	6.4	4,910	2.0	70	5.1	1,887	1.3	60	6.9	1,592	1.9	43	6.1	1,608	1.8
Reproductive	1,796	83.3	194,206	80.9	1,079	79.2	105,689	75.2	687	79.3	63,910	76.1	572	81.5	72,691	81.3

ICD Codes used in classification of ED/OS utilizations

	ICD-9	ICD-10		ICD-9	ICD-10
Neuro/Psychiatric	290–319	F01-F99	Cardiovascular	390–459	I00-I99
Respiratory/Pulmonary	460–519	J01-J99	Renal/Urinary	580–629	N00-N99
Musculoskeletal	710–739	M00-M99	Hematologic	280–289	D50-D89
Gastrointestinal/Digestive	520–579	K00-K95	Reproductive	630–679	O00-O9A
Endocrine	240–279	E00-E89			

© 2024 Declercq ER et al. *JAMA Network Open*

eTable 2. SMM (per 10,000 deliveries) among those with either an Emergency Department (ED) Visit or Observational stay (OBS) using women's most recent delivery, Massachusetts (10/1/2002-3/31/2020)

	SMM 20 at delivery N (%)		SMM 20 at delivery	Crude Odds Ratio[3]		Adjusted Odds Ratio[3]	
	Yes	No		OR	95% CI	OR	95% CI
Total	5,676	768,416	73.3				
ED/OS Utilization by Women with a **Singleton Birth**							
0	3,195 (56.3)	515,108 (67.0)	61.6	ref		ref	
1	1,026 (18.1)	134,997 (17.6)	75.4	1.23	1.14-1.32	1.14	1.06-1.22
2	470 (8.3)	50,656 (6.6)	91.9	1.50	1.36-1.65	1.32	1.19-1.46
3	218 (3.8)	22,025 (2.9)	98.0	1.60	1.39-1.83	1.35	1.17-1.56
4+	290 (5.1)	24,694 (3.2)	116.1	1.89	1.68-2.14	1.50	1.32-1.71
ED/OS Utilization by Women with **Multiple Births**							
0	325 (5.7	13,384 (1.7)	237.1	ref		ref	
1	79 (1.4)	4,351 (0.6)	178.3	0.75	0.58-0.96	0.77	0.60-0.99
2	42 (0.7)	1,712 (0.7)	239.5	1.01	0.73-1.40	1.08	0.77-1.51
3	*						
4+	*						
ED/OS Utilization by Women with **No Prior SMM**							
0	3,345 (58.9)	526,699 (68.5)	63.1	ref		ref	
1	1,065 18.8)	138,711 (18.1)	76.2	1.21	1.13-1.30	1.13	1.05-1.21
2	484 (8.5)	52,089 (6.8)	92.1	1.46	1.33-1.61	1.32	1.19-1.45
3	225	22,585	98.6	1.57	1.37-1.80	1.36	1.18-1.57

© 2024 Declercq ER et al. *JAMA Network Open*

	(4.0)	(2.9)					
4+	273 (4.8)	25,240 (3.3)	107.0	1.70	1.50-1.93	1.41	1.23-1.61
ED/OS Utilization by Women with **Prior SMM**							
0	175 (3.1)	1,793 (0.2)	889.2	ref		ref	
1	40 (0.7)	637 (0.1)	590.8	0.64	0.45-0.92	0.65	0.44-0.96
2	28 (0.5)	279 (<0.1)	912.1	1.03	0.68-1.56	1.03	0.64-1.65
3	*						
4+	34 (0.6)	224 (<0.1)	1,317.8	1.56	1.05-2.30	1.17	0.72-1.90

* Less than 20 cases.

© 2024 Declercq ER et al. *JAMA Network Open*

eFigure 1. Study Sample

Supplemental Figure 1

Study Sample

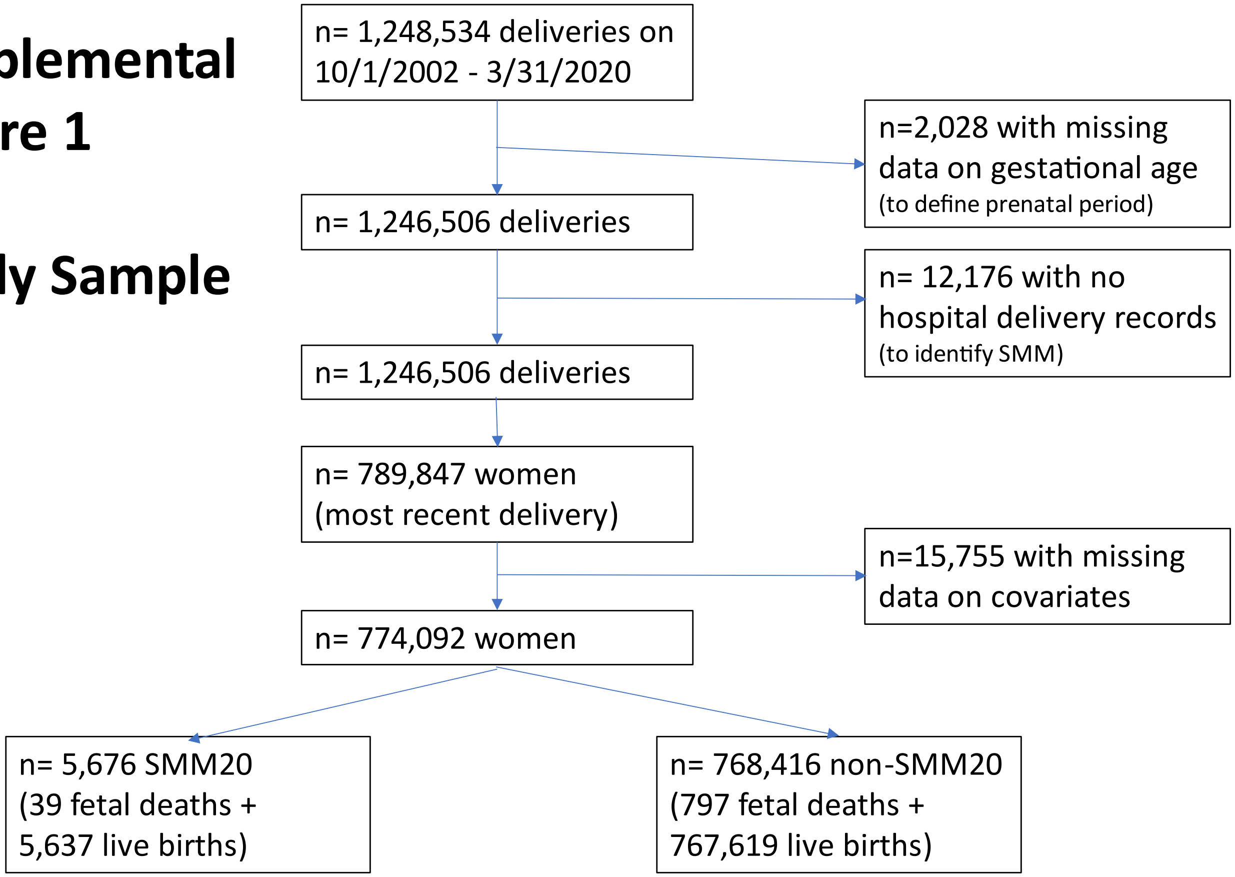

Source: PELL 1998-2020; ED data became available 1/1/2002

© 2024 Declercq ER et al. *JAMA Network Open*

eFigure 2. Timing of Unscheduled Prenatal Hospital Utilization by Prenatal Month, Massachusetts, 10/1/2002-3/31/2020

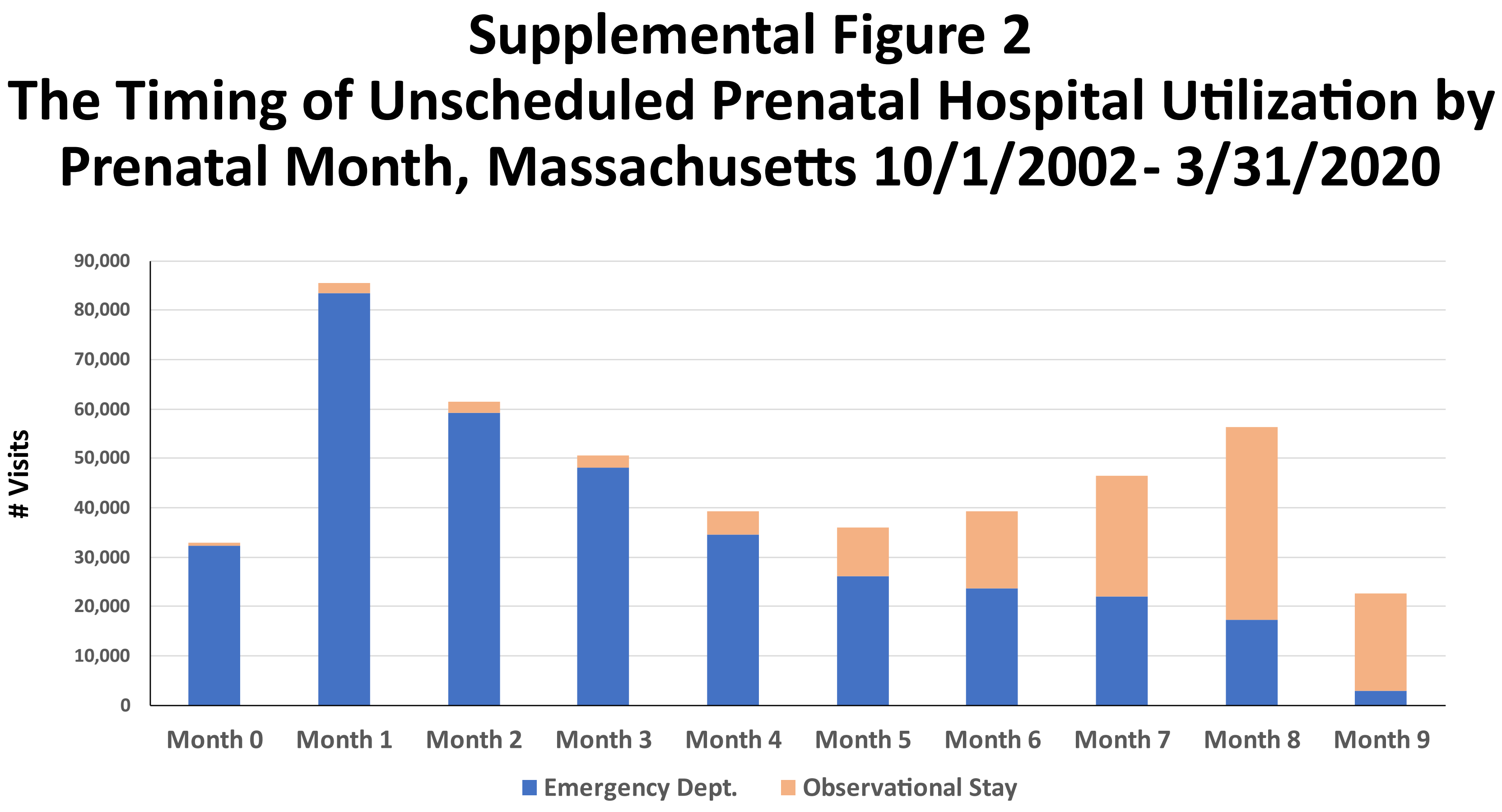

© 2024 Declercq ER et al. *JAMA Network Open*
