# Supplementary material for: Emergency Care Use During Pregnancy and Severe Maternal Morbidity
Source: JAMA Netw Open. 2024 Oct 16;7(10):e2439939. doi: 10.1001/jamanetworkopen.2024.39939 (PMC11581629; doi:10.1001/jamanetworkopen.2024.39939)
Supplement: Supplement 2. — Data Sharing Statement [file jamanetwopen-e2439939-s002.pdf]

Data Sharing Statement

Declercq. Emergency Care Use During Pregnancy and Severe Maternal Morbidity. *JAMA Netw Open*. Published October 16, 2024. doi:10.1001/jamanetworkopen.2024.39939

Data
Data available: No

Additional Information
Explanation for why data not available: Data is not available through the authors. It is only available through an application to the Massachusetts Department of Public Health IRB process for access.
